# Bee Visitation to Three Clover Species (*Trifolium* spp.) and Common Selfheal (*Prunella vulgaris*) and Establishment of These Floral Resources in Turf

**DOI:** 10.3390/insects17080792

**Published:** 2026-07-31

**Authors:** Julia J. Vieira, Casey L. Johnson, Elizabeth M. Varkonyi, Howard S. Ginsberg, Steven R. Alm

**Affiliations:** 1 Department of Plant Sciences and Entomology, University of Rhode Island, Kingston, RI 02881, USA; vieiraju23@gmail.com (J.J.V.); caseyljohnson@uri.edu (C.L.J.); elizabeth.varkonyi@dem.ri.gov (E.M.V.); hginsberg@uri.edu (H.S.G.); 2 U.S. Geological Survey, Eastern Ecological Science Center, Rhode Island Field Station, University of Rhode Island, Kingston, RI 02881, USA

**Keywords:** native bees, pollinators, bee visitation, selective foraging, floral abundance

## Abstract

The main objectives of this study were to look at bee visitation to some common forage plants that could be used in place of or in conjunction with turfgrasses and to compare establishment methods to increase pollinator forage in turfed landscapes. Bee foraging patterns were assessed on four flower species: white clover (*Trifolium repens* L.), medium and mammoth red clover (*Trifolium pratense* L.), crimson clover (*Trifolium incarnatum* L.), and common selfheal (*Prunella vulgaris* L. var *lanceolata* [Barton] Fern.) by conducting quadrat surveys on two floral plots in southern Rhode Island. Flower species were planted in randomized replicated plots to assess comparative foraging patterns of local bee species. Medium *Trifolium pratense* had the highest number and diversity of bee foragers in 2022. In 2023, *T. pratense* maintained high visitation, but the trend in diversity of visitors shifted, with *T. repens* and *P. vulgaris* having the highest bee species richness. Floral visitation generally followed shifting floral abundances, with some variation among bee species, potentially due to selective preferences. Overall, *Trifolium* spp. and *P*. *vulgaris* are valuable floral resources that support a wide range of bee species and can be included in pollinator plantings, incorporated into mowed turf areas/lawns, or used separately to enhance available bee forage. We also present results on the effectiveness of five different methods (flail mowing, flail mowing/slice seeding, rototilling, herbicide, and occultation via weed fabric) in establishing these four species as well as creeping thyme (*Thymus serpyllum* L.) in turf.

## Simple Summary

Native bees are experiencing global declines due to habitat loss, pesticide use, pathogen spread, and climate change. Turfgrass is a major component of urban landscapes, but mown turf does not offer any nectar or pollen to bees. We established two plantings of white clover, medium and mammoth red clover, crimson clover, and common selfheal which are amenable to close mowing and offer significant floral rewards to bees, and assessed bee visitation to these floral species for two years. We also evaluated various methods of establishing these floral species as well as creeping thyme in turf. These methods included flail mowing, flail mowing/slice seeding, rototilling, herbicide, and occultation via weed fabric. We found that all of the flower species were used by bees and could increase floral resources for bees wherever turf is established. We also found that bees would forage selectively on certain flowers even though other flower species were more abundant. Occultation using weed fabric or herbicides were the best treatments to establish these floral resources in turf. These floral resources could be used in conjunction with turfgrasses or used separately as alternatives to turf to increase floral resources in urban areas.

## 1. Introduction

Native bees are experiencing global declines due to factors such as habitat loss, pesticide use, pathogen spread, and climate change [1,2,3,4,5]. Comprehensive studies of bumble bee abundance and diversity in several New England states have documented declines throughout the region, including New Hampshire [6], Vermont [7], Maine [8,9], and Rhode Island [10]. Conserving native bee diversity and abundance is necessary because of their ecological and economic importance [11,12,13,14,15,16,17,18,19,20,21,22]. As native bee decline is becoming more well-documented, there are increasingly more local campaigns to plant flowers to support bee and other pollinator populations. This study aims to explore how local conservation initiatives can be improved by identifying floral preferences and other factors that affect bee visitation patterns.

For this study, floral species were selected based on their observed attractiveness to several bee taxa, ability to establish in turf, and amenability to mowing. Approximately 40 million acres of the United States is covered by lawn turf [23]. Converting turf lawns into more pollinator-friendly plantings can increase floral availability and create habitat corridors that connect green spaces in the landscape [24,25]. Doing so may help to reduce the negative impacts of habitat fragmentation and decrease inputs such as water, fertilizers, and pesticides into these areas [26], thereby supporting pollinator health. If turf areas are to be modified to support bee populations, it is important for the incorporated floral resources to withstand repeated mowing throughout the year. For this reason, we selected *T. repens*, medium and mammoth *T. pratense*, *T. incarnatum*, and *P. vulgaris* as they are often found growing and blooming in maintained lawns that undergo frequent mowing.

The first objective of this research project was to survey the abundance, diversity, and floral preferences of honey, bumble, and other wild bees to several flower species in Rhode Island: white clover (*Trifolium repens* L.), medium and mammoth red clover (*Trifolium pratense* L.), crimson clover (*Trifolium incarnatum* L.), and common selfheal (*Prunella vulgaris* L. var *lanceolota* [Barton] Fern.). Floral preference is the probability of a bee selecting a plant species regardless of its density [27] and can also be impacted by resource availability. Localized foraging patterns are important to consider in conservation programs because floral preferences can differ amongst local populations. Several factors play a role in driving foraging patterns, including variations in bee body size and proboscis length resulting in “majoring” and “minoring” observed in bumble bees [28], spatial variation in pollinator communities and flowering plants [29], phenological differences between plants and pollinators [30], pollen nutritional content [31,32,33], the timing of floral nectar and pollen production [13], and nectar quality [34]. Thus, floral visitation patterns can change across a species’ range, resulting in a “geographic mosaic” of floral preferences within a single bee species population [35]. Shifting management actions to target localized foraging patterns will therefore improve the benefit of pollinator-friendly plantings to local bee populations.

The second objective of this study was to determine the best method of establishing these four species and *Thymus serpyllum* L. in turf. *Thymus serpyllum* is another lawn alternative that is also amenable to mowing and is attractive to several bee taxa [36]. Floral abundance surveys were conducted to assess the establishment of these forbs in turf. Our findings provide recommendations for the creation and enhancement of pollinator-friendly green spaces.

## 2. Materials and Methods

### 2.1. Study Site

All aspects of this research project were conducted at the University of Rhode Island’s East Farm Agricultural Experiment Station in Kingston, RI, USA.

### 2.2. Seed Sources

*Trifolium* seeds (*T. repens*, medium *T. pratense*, mammoth *T. pratense*, and *T. incarnatum*) and *Thymus serpyllum* seeds were purchased from Johnny’s Selected Seeds (Waterville, ME, USA). *Prunella vulgaris* var *lanceollata* seeds were purchased from Silver Falls Seed Company (Silverton, OR, USA).

### 2.3. Site Preparation

Plot A (12 × 30.5 m) was established in a sandy loam soil with a pH of 5.8 and medium-high organic matter, very low nitrate nitrogen and ammonium nitrogen, medium phosphorous, low potassium, low calcium, and medium magnesium (The Connecticut Agric. Exp. Station soil testing lab). In July 2021, the plot was seeded with a rate of 56 kg/ha Royal Wing black oil sunflower wild bird seed (Tractor Supply Co., Brentwood, TN, USA) to act as a smother crop for weed control. On 14 March 2022, the sunflower stalks were rototilled, and the area was divided into twenty 3 × 6.1 m subplots (five treatments × four replicates in a randomized complete block design) and seeded with medium and mammoth *T. pratense* (2.237 g/m^2^), *T. incarnatum* (2.806 g/m^2^), *T. repens* (1.124 g/m^2^), and *P. vulgaris* (0.002 g/m^2^) using 355 mL glass cheese shakers. EXCEED alfalfa/true clover inoculant (Johnny’s Selected Seeds, Waterville, ME, USA) was added to the *Trifolium* seed prior to treatment at a rate of 7.5 g/kg of seed. The four corners of each subplot were marked with 1.2 m orange fiberglass driveway poles made with orange reflective fiberglass (ZNCMRR, Amazon.com, Inc., Seattle, WA, USA).

Plot B (24 × 30.5 m) was established in a sandy loam soil with a pH of 5.5 and medium-high organic matter, very low nitrate nitrogen and ammonium nitrogen, low phosphorous, low potassium, low calcium, and medium low magnesium (The Connecticut Agric. Exp. Station soil testing lab). On 17 June 2022, the plot was rototilled and seeded with the five flower treatments. Three years of buckwheat (*Fagopyrum esculentum* Moench) plantings (101.1 kg/ha) preceded treatment, and buckwheat stalks were rototilled into the soil prior to seeding the test species. Eight replicates of each species were established on this site in a randomized complete block design. The four corners of each subplot were marked with 1.2 m orange fiberglass driveway poles as described in Plot A.

### 2.4. Bee and Floral Abundance Surveys

Morning (10 A.M.) and afternoon (2 P.M.) bee surveys were conducted in each subplot for all species in bloom twice weekly, weather permitting from 23 June to 7 October 2022 and 24 May to 24 August 2023. Surveyors were randomly assigned to a floral treatment within the same replicate during each sample, and they collected all bees foraging on the flowers within the subplot for a total of five minutes. Surveyors walked the perimeter of the subplot to minimize disturbance and trampling of the flowers. Bees were temporarily captured in clear 50 mL numbered hinged top vials made with natural polypropylene (Thornton Plastics, Salt Lake City, UT, USA). After five minutes, surveyors identified the sex and lowest taxon possible (family/genus/species) of each captured bee. Photos and videos were taken of any bees that were difficult to identify in the field for later confirmation. Solitary bees that could not be identified to at least taxonomic family in the field were chilled, identified to genus or family under a microscope, and released thereafter. *Bombus* spp. and *Apis mellifera* were identified to species, and all other wild bees were identified at least to family. Species complexes were used for *B. vagans/sandersoni* and occasionally *B. perplexus/vagans/sandersoni* when characteristics were indistinguishable in the field. Surveyors moved sequentially through the replicates until every subplot was surveyed. Floral abundance in each subplot was also noted daily after the afternoon sampling. The number of flower heads in bloom within a 0.1 m^2^ wooden quadrat were recorded in all four corners and the approximate center of each subplot (0.5 m^2^ total). The five locations in each subplot were marked with green-colored, 2.5 cm Stake Chaser whisker marking flags (Smi-Carr, Inc., Abilene, TX, USA) to obtain floral abundance data in the same spots over the course of the season.

Due to the high abundance of small bees foraging on *P. vulgaris*, supplementary native bee surveys were conducted 21 June to 7 July 2023 to gain a better understanding of what bees apart from *Apis* and *Bombus* were using *P. vulgaris* in Plot B. The same survey methods were used, except only non-*Apis* and non-*Bombus* bees were targeted during the five-minute surveys in each *P. vulgaris* replicate.

### 2.5. Methods of Establishment Floral Abundance Surveys

**Figure 1:**
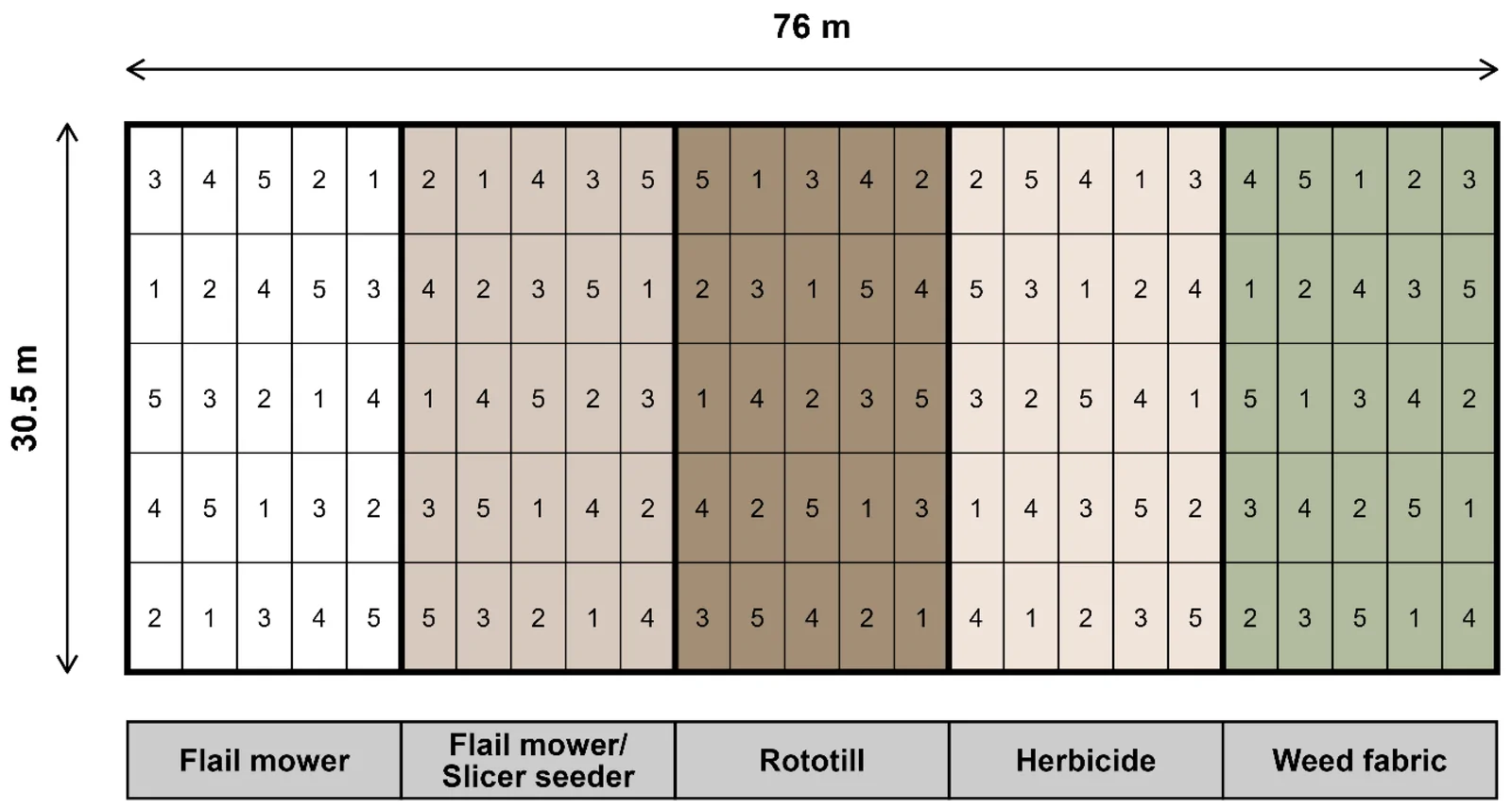
Map of the methods of establishment plot showing the five treatment areas and the randomized floral species: (1) *Thymus serpyllum*, (2) medium *Trifolium pratense*, (3) *T. incarnatum*, (4) *T. repens*, (5) *Prunella vulgaris*. Each floral species has five replicates within each treatment area.

A 76 × 30.5 m plot was established in a sandy loam soil with a pH of 6.2 and medium-high organic matter, very low nitrate nitrogen and ammonium nitrogen, medium low phosphorous, medium potassium, high calcium, and medium magnesium (The Connecticut Agric. Exp. Station soil testing lab). The main plot was divided into five 15.2 × 30.5 m treatment areas. The treatments consisted of various methods of establishing flower seed in turf, which included flail mowing, flail mowing/slice seeding, rototilling, herbicide, and occultation via weed fabric (Figure 1). Five floral species (seeding rate): *T. serpyllum* (0.067 g/m^2^), medium *T. pratense* (2.26 g/m^2^), *T. incarnatum* (2.79 g/m^2^), *T. repens* (1.13 g/m^2^), and *P. vulgaris* 0.699 g/m^2^) were randomly assigned to twenty-five 3 × 6.1 m subplots in each treatment area. *Thymus serpyllum* was seeded at three times the recommended seeding rate because the small seed size made even seeding difficult. Weed fabric (GCB, Item #78-2440, 4.7 × 91.4 m, Lumite Inc., Alto, GA, USA) was placed and secured with 15.24 cm sod staples (Griffin Greenhouse Supplies, Tewksbury, MA, USA) on 8 October 2022. Cornerstone Plus herbicide (Glyphosate 41.0%, Winfield Solutions, LLC, St. Paul, MN, USA) was applied on 15 October 2022. Rototilling was conducted on three occasions: 22 October 2022, 7 November 2022, and 21 November 2022. Flail mowing was conducted on 12 November 2022. A slice seeder with an empty hopper was used on 27 November 2022. The edges of each treatment area were marked with orange fiberglass driveway poles, and the four corners of each subplot were marked with 0.4 m Presco colored stake wire marking flags (Forestry Suppliers, Inc., Jackson, MS, USA). On 28 November 2022, the weed fabric was removed along with the remaining dead organic matter. Following the removal of the organic matter, the five floral species were seeded using glass cheese shakers. Five replicates of each floral species were established in each of the treatment areas on the site.

In May 2023, moderate to high grass and invasive weed pressure was noted. *Rosa multiflora* Thunburg and other woody plants were removed with shovels prior to mowing all five treatment areas to prevent grasses from going to seed. Floral abundance counts were conducted throughout all five treatment areas on 21 June, 3 and 13 July 2023 following the methods previously described for “Bee and floral abundance surveys”.

### 2.6. Statistical Analysis

Potential differences in bee visitation to various flower species were tested by chi square analyses, SAS 9.4, FREQ procedure [37] using results from four combined replicates in Plot A and eight combined replicates in Plot B of the four flower species (three *Trifolium* spp. and *P. vulgaris*). We tested whether the different bee taxa foraged equally on different flower species each month using tables with columns of bee taxa vs. rows of floral species. The entries were the numbers of visits by each bee taxon to each flower species. We assessed the number of visits per flower by each bee species using ANOVA, SAS 9.4, GLM procedure [37]. The dependent variable was the number of visits per flower of each bee species for each flower species (log transformed to stabilize trends in residuals), and the independent variables included the classification variables “bee species” and “flower species” with an interaction term. A significant interaction between bee species and flower species indicated selective foraging on flower species by the various bee species. Finally, we tested whether a given bee taxon foraged on various flower species in proportion to floral abundance with chi square analyses using tables with two columns (total numbers of bee visits and numbers of inflorescences) vs. rows of floral species. All figures were created in R version 4.6.0 [38,39,40].

## 3. Results

During months of greatest foraging activity, abundant bee species differed in their utilization patterns of available flowers (Table 1). Furthermore, individual bee species generally foraged selectively, in that their utilization of available flower species differed from the relative abundances of the flower species (Table 2). Details are provided below.

### 3.1. Bee and Floral Abundance Surveys (23 June to 7 October 2022)

**Figure 2:**
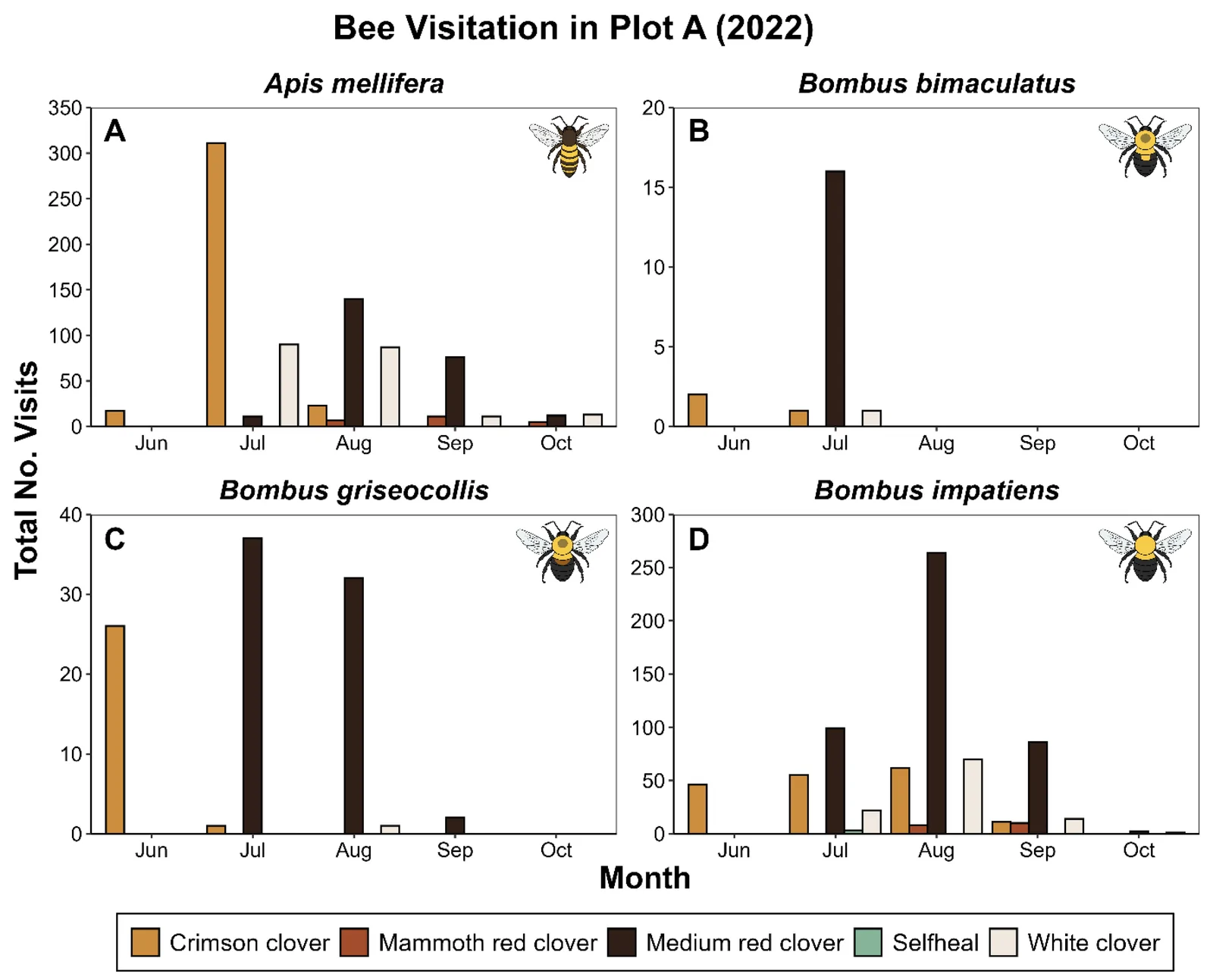
Total number of (**A**) *Apis mellifera*, (**B**) *Bombus bimaculatus*, (**C**) *B. griseocollis*, and (**D**) *B. impatiens* visiting each floral species per month in Plot A, 2022.

In 2022, a total of 1853 bees were observed in Plot A (Supplementary Files). Visitation numbers of different bee species varied on the four flower species in July (X^2^ = 369.921, df = 8, *p* < 0.0001). Visitation by different bee species varied on the four flower species in July (interaction of bee species*flower species, *F* = 7.71, df = 10.54, *p* < 0.0001). In July, *Apis mellifera* foraged selectively (X^2^ = 153.318, df = 2, *p* < 0.0001), mostly on *T. incarnatum* and *T. repens* (Figure 2A), and *Bombus bimaculatus* foraged selectively (X^2^ = 27.557, df = 2, *p* < 0.0001), mostly on medium *T. pratense* (Figure 2B).

**Figure 3:**
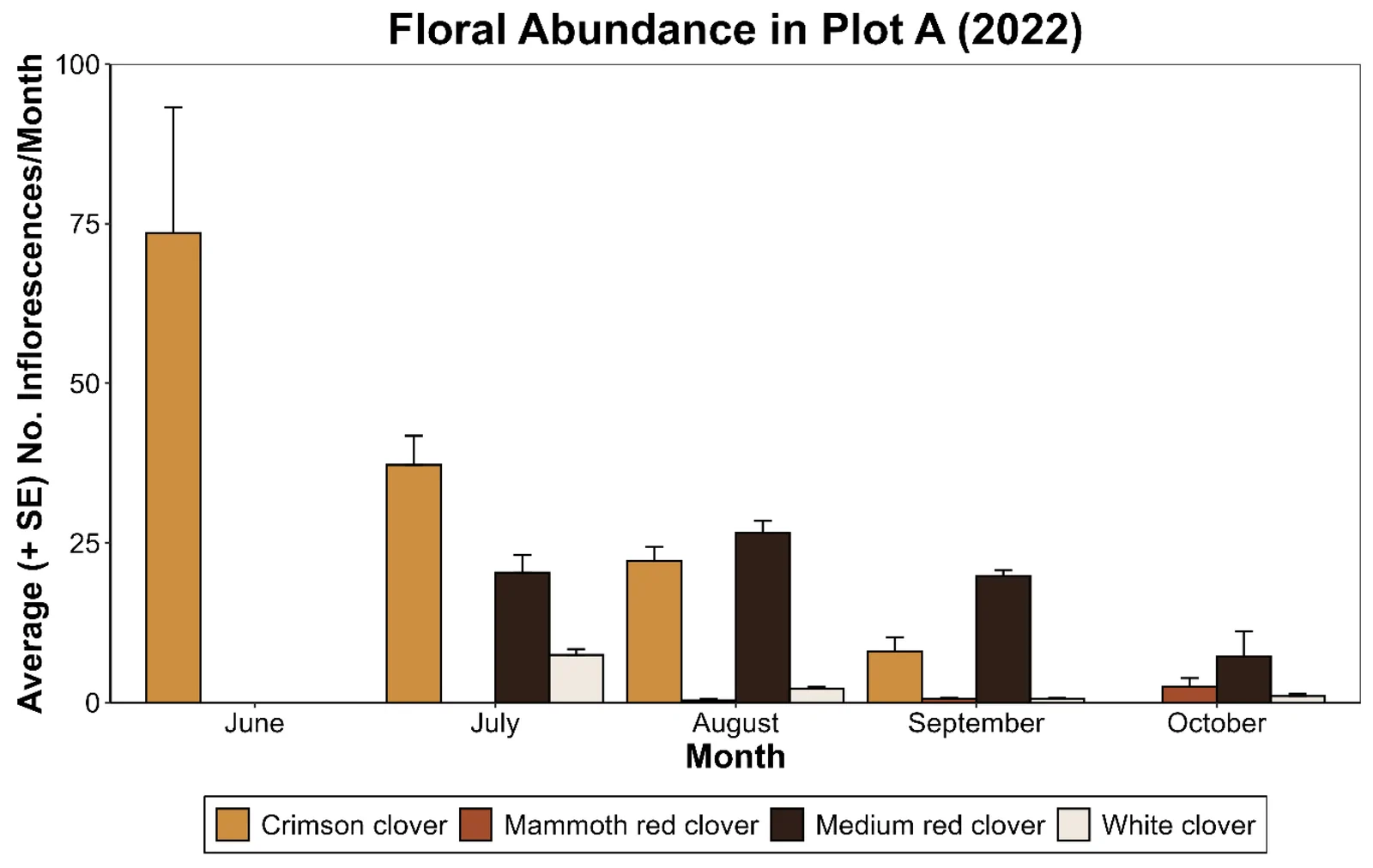
Floral abundance per month in Plot A, 2022.

The number of visits per flower differed among bee species for the different flower species in August (interaction of bee species*flower species, *F* = 12.66, df = 9,48, *p* < 0.0001). In August, both *A. mellifera* (X^2^ = 344.260, df = 3, *p* < 0.0001) (Figure 2A) and *Bombus impatiens* (X^2^ = 182.855, df = 3, *p* < 0.0001) (Figure 2D) foraged selectively on the four clover taxa. Different bee species also foraged in different numbers on the four flower species in August (X^2^ = 85.199, df = 9, *p* < 0.0001). In general, the greatest visitation was by *B. griseocollis* and *B. impatiens* to medium *T. pratense* over the season (Figure 2C,D), even though medium *T. pratense* was not always the most abundant floral species (Figure 3). *Apis mellifera*, *B. griseocollis*, and *B. impatiens* foraged abundantly on medium *T. pratense* in August (Figure 2A,C,D), which had the highest average floral abundance at this time (Figure 3), and both *A. mellifera* and *B. impatiens* also foraged abundantly on *T. repens* in August (Figure 2A,D), which had relatively low floral abundance (Figure 3). *Bombus fervidus*, an IUCN (International Union for Conservation of Nature) Red List Vulnerable species [41], was collected on medium *T. pratense*, *T. incarnatum*, and *T. repens*, but only 6 individuals were observed, so this species was not included in the statistical analysis.

In September, both *A. mellifera* (Figure 2A) and *B. impatiens* (Figure 2D) foraged selectively on medium *T. pratense* (X^2^ = 9.717, df = 3, *p* = 0.021), which had the highest floral abundance at this time (Figure 3).

### 3.2. Bee and Floral Abundance Surveys (24 May to 24 August 2023)

**Figure 4:**
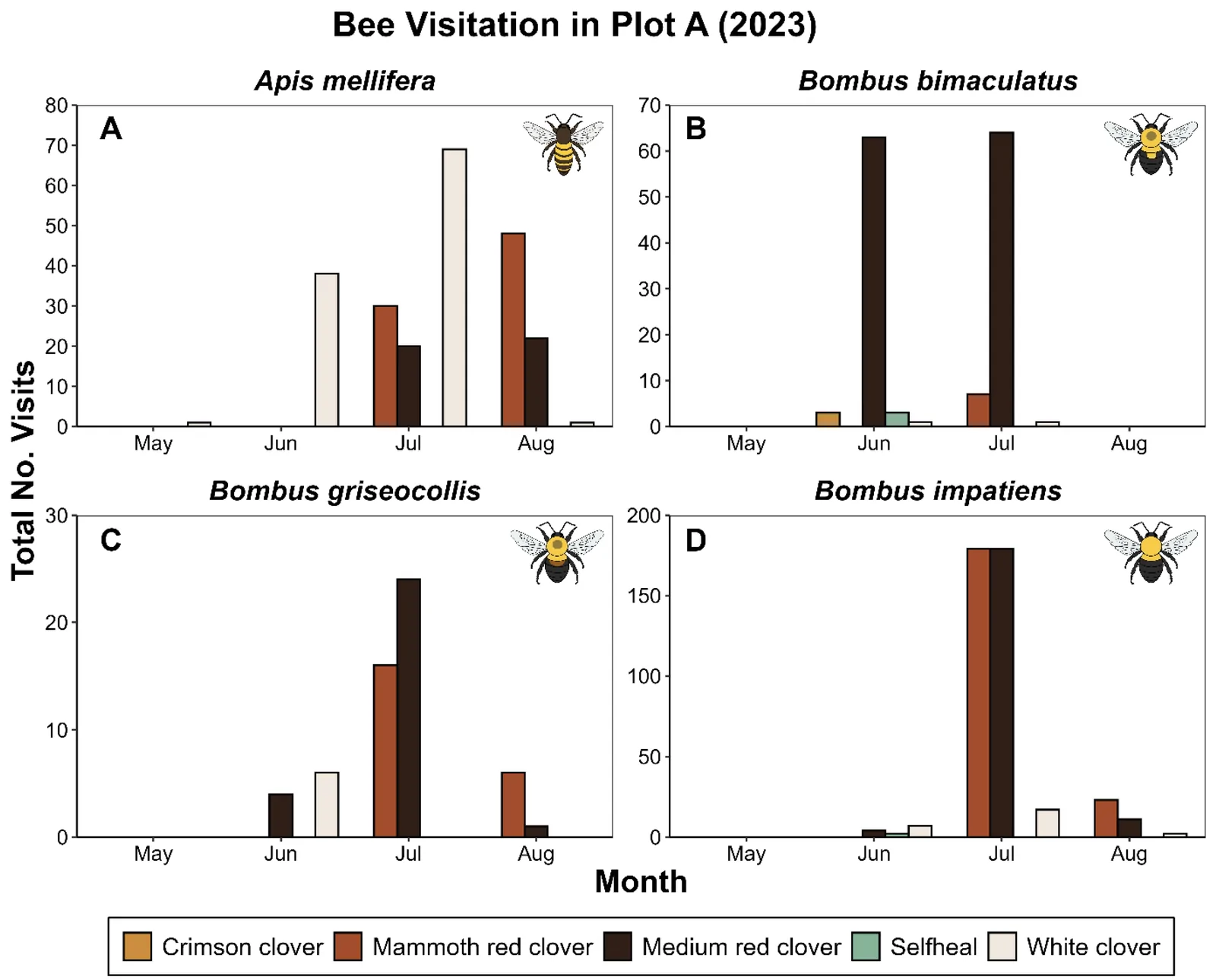
Total number of (**A**) *Apis mellifera*, (**B**) *Bombus bimaculatus*, (**C**) *B. griseocollis*, and (**D**) *B. impatiens* visiting each floral species per month in Plot A, 2023.

**Figure 5:**
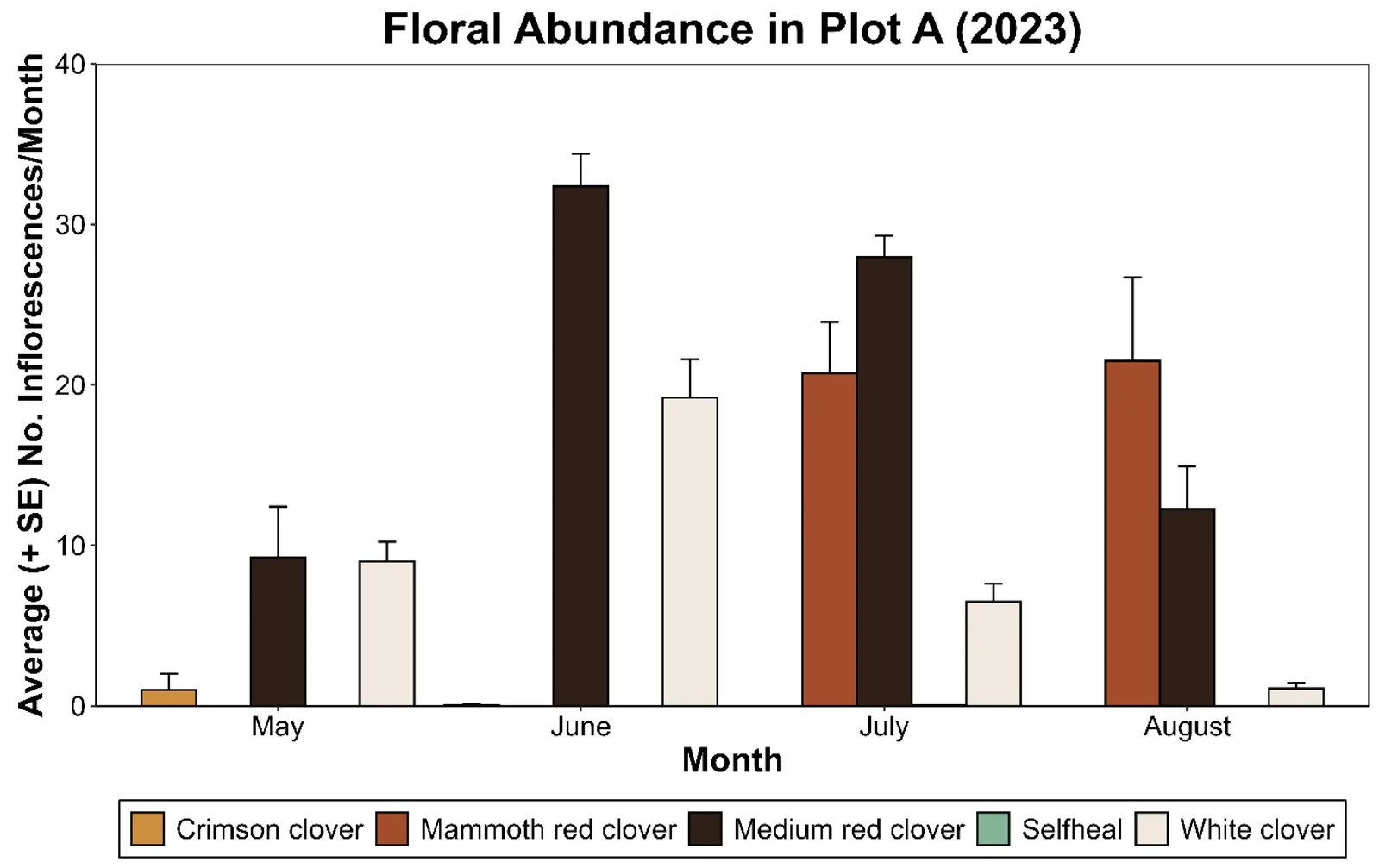
Floral abundance per month in Plot A, 2023.

In 2023, a total of 1046 bees were observed in Plot A. *A. mellifera* foraged predominantly on *T. repens* in both June and July (Figure 4A), even though medium *T. pratense* was more abundant (Figure 5). Medium *T. pratense* had the highest number of total observations (476), with *B. impatiens* having the highest number of total visitations (Figure 4D). Bee taxa differed in the number of visits per flower to different flower species (*F* = 17.33, df = 12,60, *p* < 0.0001) in June. *Bombus bimaculatus* foraged mostly on medium *T. pratense* (Figure 4B), which had the highest average floral abundance at this time (Figure 5). In contrast, *B. griseocollis* mostly foraged on *T. repens* in June, but then switched to mammoth and medium *T. pratense* in July and August (Figure 4C). *P. vulgaris* had poor germination in Plot A in both survey years, and *T. incarnatum* had poor germination in Plot A in 2023, so there were few observations of bees on these flower species in their respective poor germination years.

**Figure 6:**
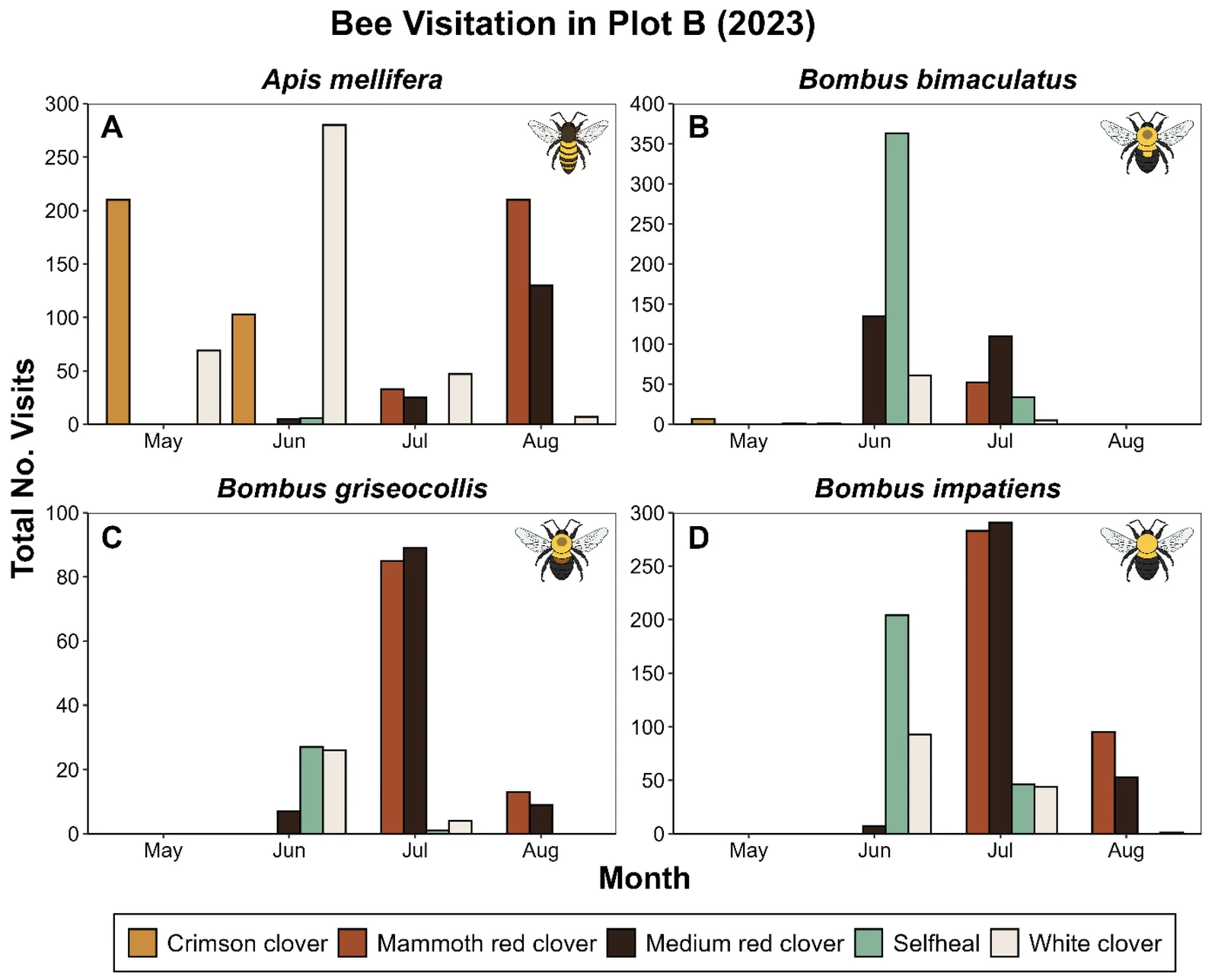
Total number of (**A**) *Apis mellifera*, (**B**) *Bombus bimaculatus*, (**C**) *B. griseocollis*, and (**D**) *B. impatiens* visiting each floral species per month in Plot B, 2023.

**Figure 7:**
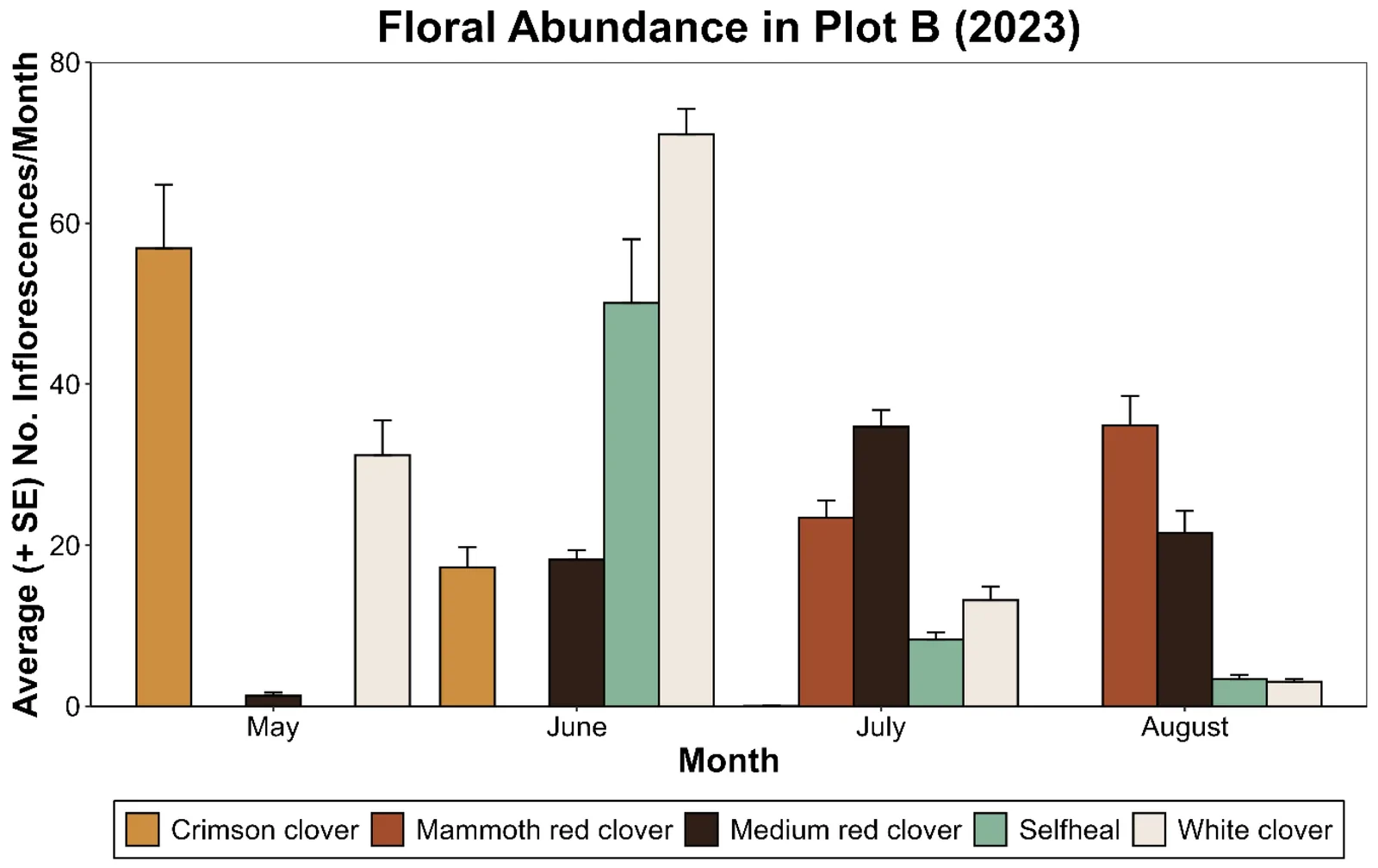
Floral abundance per month in Plot B, 2023.

In Plot B, a total of 3849 bees were observed, and all four floral species germinated well. Medium *T. pratense* had the highest number of total observations (978), followed by *T. repens* (880), mammoth *T. pratense* (824), *P. vulgaris* (804), and *T. incarnatum* with the lowest number of visitations (363). Abundant bee taxa (Andrenidae, *A. mellifera*, *B. bimaculatus*) did not differ significantly in floral visitation (X^2^ = 2.849, df = 2, *p* = 0.241) in May, although *A. mellifera* foraged selectively (X^2^ = 11.015, df = 2, *p* = 0.0041), mostly on *T. incarnatum* (Figure 6A), which was the most abundant flower at this time (Figure 7). In June, abundant bee taxa (Andrenidae, *A. mellifera*, *B. bimaculatus*, *B. griseocollis*, *B. impatiens*, and *B. vagans/sandersoni*) differed significantly in overall floral visitation (X^2^ = 773.251, df = 10, *p* < 0.0001). The number of visits per flower for each flower species differed among bee species (*F* = 8.20, df = 19,130, *p* < 0.0001). *Andrena* spp. (X^2^ = 36.162, df = 3, *p* < 0.0001), *A. mellifera* (X^2^ = 283.561, df = 3, *p* < 0.0001), and *Bombus* spp. (X^2^ = 542.304, df = 3, *p* < 0.0001) foraged selectively, and generally differed in floral species visited (Figure 6A–D and Figure 7). In a similar fashion, the number of visits per flower by each bee species for each flower species differed significantly in July (*F* = 8.00, df = 19,140, *p* < 0.001). *A. mellifera* (X^2^ = 49.323, df = 2, *p* < 0.0001), *B. bimaculatus* (X^2^ = 37.337, df = 3, *p* < 0.0001), *B. griseocollis* (X^2^ = 58.149, df = 3, *p* < 0.0001), *B. impatiens* (X^2^ = 73.219, df = 3, *p* < 0.0001), and *B. vagans/sandersoni* (X^2^ = 24.774, df = 3, *p* < 0.0001) foraged selectively and generally differed in floral species visited (Figure 6A–D and Figure 7). *A. mellifera* again foraged relatively abundantly on *T. repens* in July (Figure 6A), even though flowering of this species had declined substantially from June (Figure 7). The number of visits per flower by each bee species for each flower species also differed significantly in August (*F* = 18.38, df = 11,84, *p* < 0.0001). However, *A. mellifera* (X^2^ = 2.965, df = 2, *p* < 0.2271), *B. griseocollis* (X^2^ = 0.069, df = 1, *p* < 0.7918), and *B. impatiens* (X^2^ = 4.394, df = 2, *p* < 0.1111) did not forage selectively in August (Figure 6A,C,D and Figure 7). *Bombus vagans/sandersoni* did forage selectively in August (X^2^ = 3.989, df = 1, *p* < 0.0458) and were observed most often on medium *T. pratense* at this time.

A total of 46 non-*Apis* and non-*Bombus* bees across eight different bee taxa were observed on *P. vulgaris* from 21 June to 7 July 2023 during targeted surveys (Table 3). The greatest number of bees were observed from the family Halictidae.

### 3.3. Methods of Establishment Floral Abundance Surveys

*Trifolium repens* and *P. vulgaris* produced more inflorescences than medium *T. pratense* and *T. incarnatum* across all treatment areas (Table 4). *T. serpyllum* bloomed later than the other flower species but also produced more inflorescences than medium *T. pratense* and *T. incarnatum* by the third survey day (Table 4). The flail mowing and flail mow/slice seeder treatments had the lowest number of inflorescences out of all treatments, with 177 and 109 inflorescences in total, respectively, while the herbicide treatment had the highest sum of inflorescences on each survey day: 277, 562, and 403 (Table 4). *P. vulgaris* had the highest floral abundance overall, with 2080 total inflorescences observed over the three survey days.

## 4. Discussion

*Trifolium pratense*, *T. repens*, *T. incarnatum*, and *P. vulgaris* are valuable floral resources that support a variety of bee taxa. In 2022, medium *T. pratense* had the highest number and diversity of bee visitors. Other insect pollinators, including monarch butterflies, swallowtails, and flower flies, were also noted foraging on medium and mammoth *T. pratense*. *P. vulgaris* and *T. repens* had the highest number and diversity of bee taxa and the highest average floral abundances in June 2023. Flower flies were also often noted foraging on *P. vulgaris*.

In Plot A (2022), *Bombus* spp. selectively foraged on medium *T. pratense* in July (Figure 2B–D and Figure 3), and in Plot B (2023), they selectively foraged on *P. vulgaris* in June (Figure 6B–D and Figure 7), when neither of these species were the most abundant. This suggests that *Bombus* spp. exhibited preferences for these flowers independent of their abundances. *A. mellifera* foraged abundantly on medium *T. pratense* and *T. repens* in Plot A in August 2022 (Figure 2A) when medium *T. pratense* was the most abundant floral species (Figure 3). This might indicate that *A. mellifera* exhibited a preference for *T. repens* at this time. In contrast, *A. mellifera* selectively foraged on *T. incarnatum*, the most abundant floral species, in Plot B in May 2023 (Figure 6A and Figure 7). Indeed, *A. mellifera* generally foraged on *T. incarnatum* and on *T. repens* to a greater extent than *Bombus* spp., which foraged to a greater extent on medium and mammoth *T. pratense*, suggesting broad differences in floral preferences. For example, *T. incarnatum* was the only flower species available in Plot A in June 2022 (Figure 3), and all four bee species foraged on that plant (Figure 2A–D). When medium *T. pratense* bloomed in July, *Bombus* spp. shifted to that flower species, while *A. mellifera* continued to forage predominantly on *T. incarnatum* (Figure 2A). *A. mellifera* foraged abundantly on *T. repens* in August 2022 (Figure 2A) and in July 2023 (Figure 4A and Figure 6A), even though *T. pratense* was more abundant, suggesting a possible floral preference. It is plausible that the smaller floret size of *T. repens* might contribute to this pattern as it would allow for easier nectar foraging by *A. mellifera* due to its shorter tongue length than most *Bombus* species.

Our results support other studies that have found *Trifolium* spp. to be an important forage plant for several *Bombus* species. Jacobson et al. [6] found that *T. pratense* and *T. repens* were particularly important to long-tongued bumble bees, and *Trifolium* spp. were among the most common forage plants in bumble bee roadside surveys in Vermont [7]. Similarly, Larson et al. [42] found that *T. repens* supported a variety of bee taxa, with *A. mellifera* being one of its most frequent visitors. Wolfin et al. [36] also reported high *P. vulgaris* floral abundance and high bee diversity on *P. vulgaris* and *T. repens* at their study sites.

The reasons for these foraging patterns might include actual floral preferences by different bee species. Preferences can result from differences among flower species in floral structure and in the production of nectar and pollen [43]. Furthermore, nectar produced by various species differs in levels of sugars, amino acids, and other possible nutrients [44]. Lun et al. [45] found that bumble bee foraging patterns were influenced by flower corolla length, nectar sugar concentration, and floral abundance. However, in our study, several other factors might have contributed to floral foraging patterns. For example, Plot B was twice the size of Plot A. It is also possible that there is more nesting habitat around Plot B, and therefore a greater variety and total number of bee taxa than in Plot A, which may have contributed to the higher number of bee observations in Plot B overall. Native bees have shorter foraging distances [46,47] than *A. mellifera*, so they will preferentially forage on plants that are in closer proximity to their nesting sites. There was also a difference in bloom start dates and months surveyed between study years. Flowers began blooming on 23 June 2022 in Plot A and on 31 May and 25 May 2023 in Plots A and B, respectively, resulting in an extra month of observations in the spring of 2023. Furthermore, Plot A was surveyed from 23 June 2022 to 7 October 2022, and Plots A and B were surveyed from 24 May 2023 to 24 August 2023. Thus, the changes in species richness and relative abundance of bee species over time could also account for the differences in bee visitation between survey years. There may have also been a difference between the timing of the pollen and nectar presentation in the flowers between survey years, which could have contributed to the differences in foraging patterns between species.

Several factors may have contributed to the poor germination of *P. vulgaris* (2022 and 2023) and *T. incarnatum* (2023) in Plot A. Native perennials often require cold/moist stratification for proper germination, and the Plot A seeding date of 14 March 2022 may not have provided enough of a cold stratification period for *P. vulgaris* to germinate prior to the first survey season. Differences in microclimates or soil moisture may have also had an impact. *Trifolium* spp. have been documented as being intolerant to frost [48], thus the February 2023 cold snap in New England [49] may have prevented *T. incarnatum* from germinating that year. Although the soil types and nutrient analyses were very similar between Plot A and Plot B, there were slight differences (pH 5.8 versus 5.5, phosphorus 25 versus 12 ppm, and magnesium 37 versus 18 ppm respectively) due to differences in site management. Seeding in Plot B was preceded by three years of buckwheat plantings, whereas seeding in Plot A was preceded by only one year of sunflower plantings. Buckwheat increases soil phosphorus and reduces soil compaction [50], whereas sunflowers do not contribute soil nutrients but are good nitrogen scavengers and have long taproots that help with soil compaction [51].

Our results contribute to the conversation about planting nonnative vs. native floral species to enhance bee forage. *Trifiolium pratense*, *T. incarnatum*, and *T. repens* are not native to North America, whereas *P. vulgaris* var. *lanceolotta* is native to Rhode Island [52]. When *P. vulgaris* flowered in Plot B in 2023 (Figure 7), it was abundantly utilized by *Bombus* spp. (Figure 6). Gegear and Laverty [53] found that bumble bees exhibit greater flower constancy in flowers that vary in multiple floral traits: color, corolla size, odor, and morphology. This may explain the possible preference displayed by bumble bees in Plot B, as *P. vulgaris* differs in both color and morphology from the *Trifolium* spp. Although *P. vulgaris* had the highest number of individual bee observations in 2023, the *Trifolium* spp. were also visited by a high number and diversity of bee taxa during both survey years. Furthermore, *Trifolium* spp., especially *T. pratense* and *T. repens*, provide long-lasting forage with blooms lasting for months. Nectar quality and quantity, and ease of access, might also contribute to their visitation. In combination, all *Trifolium* spp. and *P. vulgaris* appear to be valuable floral resources to add to bee-friendly lawns and pollinator plantings.

The low overall establishment of plants across all five treatments in the methods plot supports the difficulty of converting turf to forbs. Turfgrass grew back and competed with the floral species in all treatment areas, although the herbicide treatment had the least amount of turfgrass regrowth over the season. The flail mowing and flail mowing/slice seed treatments were not as effective in establishing the five floral species as the rototill, herbicide, and occultation treatments. These two treatments likely did not provide enough seed to soil contact for proper germination. Both the rototill and occultation treatments had a moderate overall floral abundance, but rototilling resulted in the greatest weed plant pressure compared to the other treatments. Although the herbicide treatment had the highest overall floral abundance, many herbicides can be lethal to bees [54,55] and can impact non-target floral resources that may provide additional forage [54,56]. Therefore, occultation with weed fabric seems to be the most environmentally desirable method for florally enhancing turf for pollinators. This study only assessed the plot one year after establishment, so future research on methods of establishing floral resources in turf over a longer period of time would be beneficial. Furthermore, future studies should consider assessing the impacts of a mowing regime on various establishment methods.

Our research improves on the current understanding of important host plants for some Rhode Island bees. Observations of floral visitation patterns on randomized plots allow for clear assessments of floral foraging patterns of different bee species. Bumble bees were shown to selectively forage on medium and mammoth *T. pratense* and *P. vulgaris* while *A. mellifera* selectively foraged on medium *T. pratense*, *T. repens*, and *T. incarnatum*. This information can be used to improve pollinator planting recommendations and other bee conservation management plans for southern New England and possibly beyond.

## Figures and Tables

**Table 1:** Comparison of foraging patterns of abundant bee species on available flowers.

Comparison	Year	Plot	June	July	August
Distribution of bees on flowers ^a^	2022	A	-	***	***
	2023	A	***	***	NS
	2023	B	***	***	NS
Bees per flower ^b^	2022	A	-	***	***
	2023	A	***	***	**
	2023	B	***	***	***

^a^ Significance of chi-square tests comparing numbers of visits of abundant bee species to available flower taxa. ^b^ Significance of interaction term “bee species * flower species” in ANOVA model with dependent variable of bee visits per inflorescence. Significance levels: NS = not significant, ** = *p* < 0.01, *** = *p* < 0.001, dash = sample size too small for statistical analysis.

**Table 2:** Selective foraging by common bee species on floral plots. Significant chi-square results indicate that bees did not visit the flowers in proportion to floral abundance (NS = not significant, *** = *p* < 0.001). Dashes indicate sample sizes too small for statistical tests.

Year	Plot	Bee Species	June	July	August
2022	A	*A. mellifera*	-	***	***
		*B. impatiens*	-	***	***
		*B. griseocollis*	-	***	-
		*B. bimaculatus*	-	***	-
2023	A	*A. mellifera*	***	***	***
		*B. impatiens*	-	***	NS
		*B. griseocollis*	-	NS	-
		*B. bimaculatus*	***	***	-
	B	*A. mellifera*	***	***	NS
		*B. impatiens*	***	***	NS
		*B. griseocollis*	NS	***	NS
		*B. bimaculatus*	***	***	-

**Table 3:** Number of bee taxa other than *Apis* and *Bombus* observed during the targeted bee surveys (21 June to 7 July 2023) on *Prunella vulgaris* plots.

Bee Taxon	No. Observed
*Agapostemon* spp.	3
Andrenidae	2
*Andrena* spp.	2
*Ceratina* spp.	23
Halictidae	37
*Halictus* spp.	6
*Hylaeus* spp.	1
*Osmia* spp.	2

**Table 4:** Total number of inflorescences in the methods of establishment plot for three survey days.

Total No. of Inflorescences						
	Flail Mow	Flail Mow/Slice Seed	Rototill	Herbicide	Weed Fabric	Grand Total
21 June 2023	62	46	31	277	157	573
*Thymus serpyllum* Creeping thyme	0	0	0	0	0	0
*Trifolium pratense* Medium red clover	0	0	0	0	0	0
*Trifolium incarnatum* Crimson clover	9	9	3	6	2	29
*Trifolium repens* White clover	52	34	2	44	20	152
*Prunella vulgaris* Selfheal	1	3	26	227	135	392
3 July 2023	83	47	399	562	349	1440
*Thymus serpyllum* Creeping thyme	0	0	14	5	5	24
*Trifolium pratense* Medium red clover	0	0	1	11	0	12
*Trifolium incarnatum* Crimson clover	3	4	9	2	1	19
*Trifolium repens* White clover	64	25	25	21	14	149
*Prunella vulgaris* Selfheal	16	18	350	523	329	1236
13 July 2023	32	16	229	403	150	830
*Thymus serpyllum* Creeping thyme	1	3	51	39	27	121
*Trifolium pratense* Medium red clover	1	0	43	56	7	107
*Trifolium incarnatum* Crimson clover	0	0	11	0	0	11
*Trifolium repens* White clover	26	13	31	34	35	139
*Prunella vulgaris* Selfheal	4	0	93	274	81	452
Grand Total	177	109	659	1242	656	2843

## Data Availability

The raw data for Figure 2, Figure 3, Figure 4, Figure 5, Figure 6 and Figure 7 can be accessed via Supplementary Files.

## Supplementary Materials

The following supporting information can be downloaded at: https://www.mdpi.com/article/10.3390/insects17080792/s1.
